# Electron attachment to fluorodeoxyglucose: Dissociation dynamics in a molecule of near-zero electron affinity

**DOI:** 10.1063/5.0101726

**Published:** 2022-08-21

**Authors:** Eugene Arthur-Baidoo, Milan Ončák, Stephan Denifl

**Affiliations:** 1 Institut fücr Ionenphysik und Angewandte Physik, Leopold-Franzens Universität Innsbruck, Technikerstrasse 25, A-6020 Innsbruck, Austria; 2 Center for Molecular Biosciences Innsbruck, Universität Innsbruck, Technikerstrasse 25, A-6020 Innsbruck, Austria

## Abstract

Fluorodeoxyglucose (FDG) is a glucose derivative with fluorine at the C_2_ position. The molecule containing the radioactive F-18 isotope is well known from its application in positron emission tomography as a radiotracer in tumor examination. In the stable form with the F-19 isotope, FDG was proposed as a potential radiosensitizer. Since reduction processes may be relevant in radiosensitization, we investigated low-energy electron attachment to FDG with a crossed electron–molecule beam experiment and with quantum chemical calculations as well as molecular dynamics at elevated temperatures to reveal statistical dissociation. We experimentally find that the susceptibility of FDG to low-energy electrons is relatively low. The calculations indicate that upon attachment of an electron with a kinetic energy of ~0 eV, only dipole-bound states are accessible, which agrees with the weak ion yields observed in the experiment. The temporary negative ions formed upon electron attachment to FDG may decay by a large variety of dissociation reactions. The major fragmentation channels include H_2_O, HF, and H_2_ dissociation, accompanied by ring opening.

## I Introduction

The role of fluorine in medicine is well established in several fields since the antineoplastic 5-fluorouracil (5-FU) was suggested as an antimetabolite.^1–4^ The small atomic radius and high electron affinity of fluorine reduce steric effects and facilitate the incorporation into drugs, activating metabolic pathways relevant to tumor-inhibiting mechanisms.^4–6^ The incorporation of fluorine derivatives, such as 5-FU and 5-fluoro-2-deoxy-uridine, into nucleosides and nucleobases of DNA and RNA has contributed to the radiosensitization of such compounds when used in cancer treatment.^1,7–10^ Modified glucose derivatives, such as 2-deoxy-D-glucose (2DG) and 2-deoxy-2-fluoro-D-glucose (FDG), have also shown potential in chemotherapy applications by demonstrating the ability to selectively block the growth pathways of cancer cells, particularly under hypoxic conditions.^3,10–13^

**Fig. 1:**
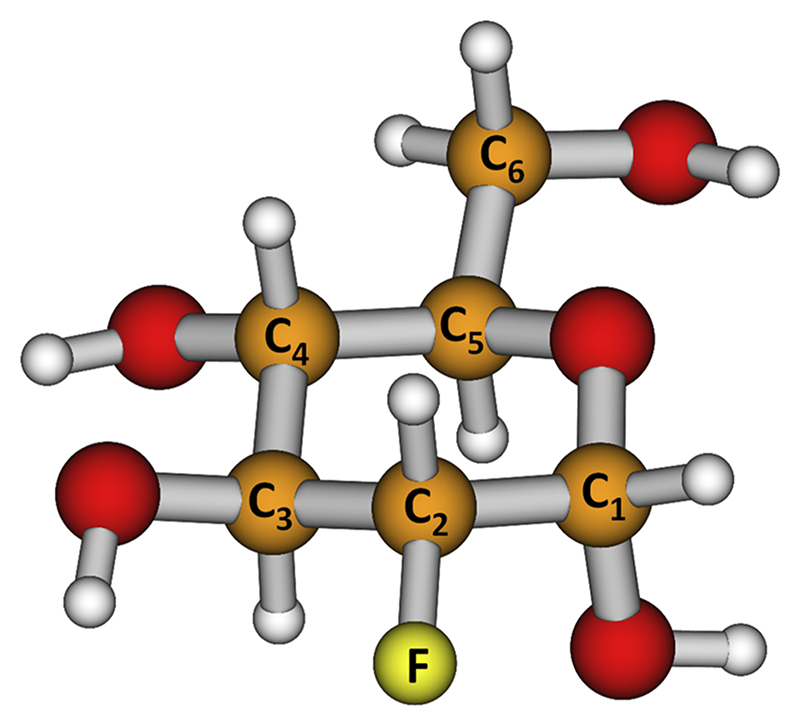
Structure of 2-deoxy-2-fluoro-D-glucose (FDG).

FDG is an analog of glucose, differing at the second carbon atom by the substitution of a hydroxyl group with a fluorine atom, as shown in Fig. 1. In nuclear medicine, its non-toxic radioactive form, 18-FDG, is used in positron emission tomography (PET) as a radiotracer in tumor examination to localize human tumors and determine the extent of disease and therapy outcomes.^10,14^ *In vitro* studies under normoxia and hypoxia showed that 19-FDG was more effective in inhibiting glycolysis and reduced cell viability than 2DG.^13^ This is linked to the fact that the chemical properties of the fluorine at the C_2_ position in FDG are more similar to that of the hydroxyl group in glucose than for the hydrogen in 2DG, which makes FDG preferable for glucose transporters.^13,15^ On the other hand, FDG is considered a better substrate because it can bind to the catalytic site of the hexokinase more efficiently than 2DG, hence the increased levels of accumulation during glycolysis.^15^

The physical mechanism supporting the radiosensitizing ability of FDG in the presence of ionizing radiation due to the role of low energy electrons (LEEs) is yet unknown. An understanding of such a mechanism is relevant to improve its activity against tumor cells and propose effective ways of drug development. LEEs with kinetic energies below 20 eV, released in large amounts per MeV of incident ionizing radiation,^16^ play a role in DNA damage.^16,17^ An underlying mechanism leading to the damage is attributed to dissociative electron attachment (DEA). DEA is a resonant process where an electron is captured by a molecule resulting in a temporary negative ion (TNI) that subsequently may decompose into a fragment anion and neutral counterpart(s). In competition to DEA, the spontaneous release of the excess electron (autodetachment) may occur.^18^ DEA in several potential radiosensitizers was investigated to reveal the formation of highly reactive radicals, fragmentation pathways, and details on reaction mechanisms.^19–23^ It has been shown studying decomposition pathways of anionic resonances observed in the gas phase may contribute to our knowledge of the mechanism in other environments.^24,25^

Due to the importance of native monosaccharides in chemistry and biology, electron attachment studies of molecules, such as deoxyribose,^26^ glucose,^27^ fructose,^27–29^ and ribose,^30^ as well as the furanose analogs furan^28^ and tetrahydrofuran,^28^ were carried out previously. A DEA study of gas-phase glucose was carried out by Shchukin and Muftakhov.^27^ Most of the negative ions were formed at electron energies below 2 eV. They documented the release of *n* H_2_O molecules, *n* = 1–3.^27^ Similarly, this behavior was also observed in the DEA study with deoxyribose by Ptasińska *et al*.^26^ Also, in this study, most of the anions were reported to be a result of loss of at least one H_2_O molecule.

In this study, we investigate electron-induced dissociation in FDG (19-FDG) upon low-energy electron attachment in the gas phase using a crossed electron-molecule beam experiment. We consider the formation of negative ions and several fragmentation pathways observed due to multiple bond cleavages within the FDG molecule. The experimental findings are supported by quantum chemical calculations that suggest possible fragmentation pathways of the anions. Computationally, we point out the role of a dipole-bound state in the electron attachment process to FDG, which acts as a doorway to dissociation reactions.

### II Experiment and Theory

DEA of FDG has been studied using a crossed electron–molecule beam experiment described in detail in Ref. 31. Herein, we present only a brief description. The setup consists of a hemi-spherical electron monochromator and a quadrupole mass analyzer for the detection of ions. The FDG sample as a powder at room temperature with a purity of 98% was purchased from Sigma-Aldrich, Austria, and used as delivered. The sample was introduced into the gas phase by a resistively heated oven. The sample was heated to a sublimation temperature of 383 K to obtain a good signal intensity while avoiding sample decomposition. To ensure this, a series of temperature-dependent positive ion mass spectra were measured starting with low oven temperatures (about 343 K). The decomposition was checked by normalizing the intensity over the parent signal and monitoring the peak ratios with varying temperatures. The decomposition was observed at the critical temperature where there is a change in the original mass spectrum. The effusive molecular beam was then introduced into the interaction region through a 1 mm capillary, which is perpendicularly crossed with a well-defined electron beam generated by a heated filament. An electron beam of full width at half maximum of 110 meV and electron current in the range of 25–80 nA was chosen as a compromise between the energy resolution and ion signal. The resulting ions were thereafter extracted by a weak electrostatic field to the entrance of the quadrupole and detected by a channel electron multiplier through a single pulse counting technique. The anionic signals were plotted as a function of the electron energy as anion efficiency curves. The energy calibration of the curves was performed using the 0 eV peak in the Cl^−^ yield of the well-known s-wave DEA reaction upon electron attachment to CCl_4_. The experiment was carried out within the electron energy range of 0–12 eV.

The initial steps after electron attachment to FDG were described using quantum chemical calculations at the B3LYP/aug-cc-pVDZ level; the local minimum or transition state character of each point was confirmed by vibrational analysis. To obtain more reliable reaction energies, the obtained structures were single-point recalculated at the Coupled Clusters Singles and Doubles (CCSD)/aug-cc-pVDZ level; the zero-point correction at the B3LYP/aug-cc-pVDZ was employed. For more robust modeling of dipole-bound states, we corrected the reaction energies by employing the aug-cc-pVDZ basis for C, O, and F and aug-cc-pVTZ for H with two additional *s* functions and one *p* and *d* function on each H atom and with basis function coefficients obtained as one-third of the lowest one for the *s, p*, and *d* functions in aug-cc-pVTZ. We denote the basis as aug-cc-pVDZ(C, O, F)TZ(H)+. The larger basis set led to no considerable changes in relative energy for structures with the odd electron localized on atoms/bonds with the average absolute difference in relative isomer energy below 0.04 eV compared to the results obtained with the aug-cc-pVDZ basis set. The correction with the aug-cc-pVDZ(C, O, F)TZ(H)+ basis was calculated at the B3LYP level and, for reaction pathways, added to the relative energy calculated at the CCSD level.

Dissociation dynamics of FDG^−^ were probed using molecular dynamics (MD) employing the semi-empirical PM6 method at the elevated temperature of 1400 K kept by the Nosé–Hoover chain thermostat. The time step of 40 a.u. was used, and the MD run was stopped in case no dissociation took place after 400 000 steps. If dissociation was observed, the fragments were recalculated at the B3LYP/aug-cc-pVDZ level to assess the dissociation energy. If the dissociation reaction was calculated to be exothermic, the neutral fragment was removed and molecular dynamics of the anionic fragment was continued. In total, 100 MD runs were started, and all channels observed in MD are collected in the supplementary material (Table S1). Note that no time evolution is addressed, and the MD approach at high temperature is only used to probe possible reaction pathways. As expected, the PM6 method cannot reproduce the results observed in the electron attachment experiment due to the complex electronic structure of the FDG anion and can be used only to assess statistical dissociation. For example, spontaneous ring opening along a C–O bond is observed at the beginning of MD runs. Still, re-calculation at a higher level of theory shows that many exothermic channels are indeed obtained, and the main experimental findings are reproduced (see below).

All quantum chemical calculations were performed in the Gaussian software,^32^ while the Abin program was used for molecular dynamics.^33^

### III Results and Discussion

**Fig. 2:**
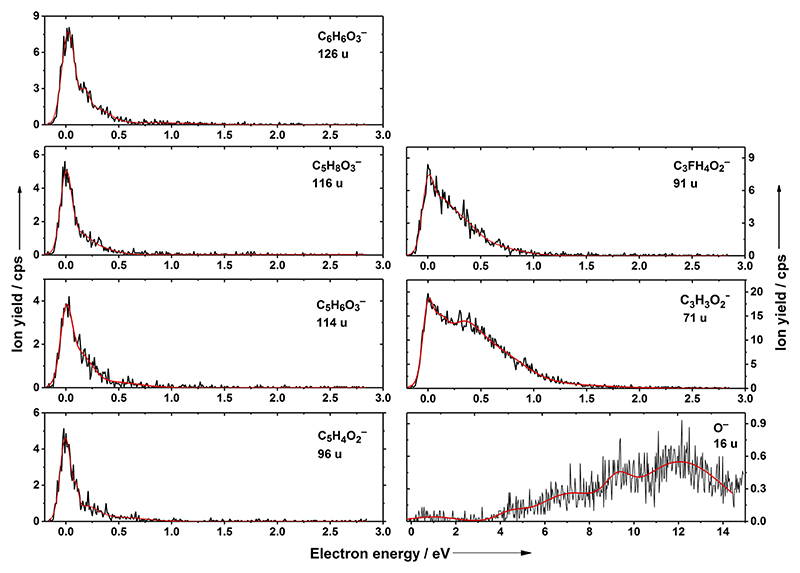
Anion efficiency curve of the anions with masses of 126, 116, 114, 96, 91, 71, and 16 u formed upon electron attachment to FDG along with their assignment based on calculations.

For DEA with FDG, we detected seven fragment anions, see Table I. The parent anion, as well as the dehydrogenated parent anion, is not observed within the detection limit of the apparatus. Remarkably, no other fragment anion formed by single bond cleavage is observed. Instead, the fragment anions measured here are due to multiple bond ruptures. Anions with masses of 12, 126, 114, and 71 u were also reported in the previous gas-phase DEA study of glucose.^27^ However, the presence of the fluorine atom opens other possible dissociation channels. The anion efficiency curves, which represent the ion yield as a function of the electron energy, are shown in Fig. 2. All fragment anions from electron attachment to FDG, except for the weakly abundant O^−^ anion, exhibit a narrow resonance close to 0 eV and other low-intensity peaks below 1 eV in the tail. The 0 eV contribution is less pronounced for the anions with masses of 91 and 71 u. This signifies that the anions are formed within the same temporary negative ion state. The O^−^ ion is mainly observed at higher electron energies, and the very low signal intensity in the 0–2 eV region can be considered as background. Although we did not estimate absolute cross-section values in this study, the rather low relative yields of all observed anions (see Fig. 2) indicate that DEA to FDG is not an efficient process compared to electron scavengers, such as tirapazamine^19,34^ or nimorazole.^20^

**Fig. 3:**
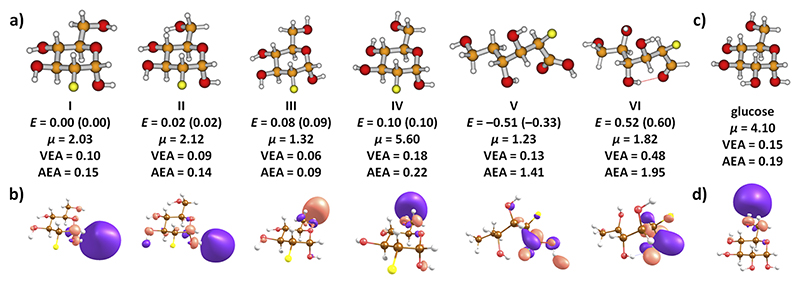
(a) Several FDG isomers along with their relative energies *E* (in eV), dipole moments *μ* (in Debye), and vertical and adiabatic electron affinity (VEA and AEA, in eV). (b) Orbitals with the odd electron after electron attachment for the respective isomers in (a). (c) and (d) A glucose isomer and the orbital with the odd electron in the respective anion. All results were calculated at the B3LYP/aug-cc-pVDZ(C, O, F)TZ(H)+//B3LYP/aug-cc-pVDZ level, see the Experiment and Theory section; values based on the CCSD level recalculation are given in parentheses.

In the neutral state, it is assumed that the FDG molecule forms a ring in the gas phase [Fig. 3(a)]. Several isomers might be localized with various relative orientations of OH groups (e.g., **I**–**IV**). These isomers are expected to interconvert at the experimental temperature; they, however, differ substantially in the dipole moment value and, thus, the ability to attract electrons. The glucose ring in the neutral FDG molecule might open in an exothermic reaction only after a proton transfer between two carbon atoms (isomer **V**). This process is, however, improbable under experimental conditions as already the breaking of the C_1_–H bond for the initial proton transfer has a barrier of ~2.4 eV as calculated at the B3LYP/aug-cc-pVDZ level.

Our calculations show that the electron affinity is sensitive to FDG conformation. For the ring structure, it is close to zero, representing a dipole-bound state [DBS, see Fig. 3(b)]. Consequently, vertical and adiabatic electron affinities (VEA and AEA, respectively) do not differ considerably. The attached electron is localized *either* next to the CH_2_OH group on an OH group close to the fluorine atom (**I, II**) or on the opposite side with respect to the fluorine atom (**III, IV**). For open structures (**V, VI**), the electron binds to the COOH or COH groups and induces further structural changes including C–F bond dissociation, reflected in the high value of adiabatic electron affinity [Fig. 3(a)].

The dipole-bound anions, such as structures **I**–**IV**, require a critical dipole moment in order to remain stable within the experimental timescales. For stationary dipoles, the limit is 1.625 D, while it increases to ~2.5 D if rotational dynamics are included.^35^ Thus, only isomer **IV** with its large dipole moment of 5.6 D may be considered as a relevant isomer for the dissociation processes discussed here. The low yields for the resonance close to ~0 eV shown in Fig. 2 (for direct DEA reactions, the cross-section would be indirectly proportional to the square root of the electron energy, and thus, a much higher ion yield would be expected^36^) also support the assumption that the FDG molecule is indeed present in its ring form in the experiment. It should be further noted that the electron is very weakly bound and, as the most probable channel, electron detachment takes place before any reaction channel could be initiated. If the dipole-bound state survives, it may act as a doorway state to dissociation processes.^29,37^

**Fig. 4:**
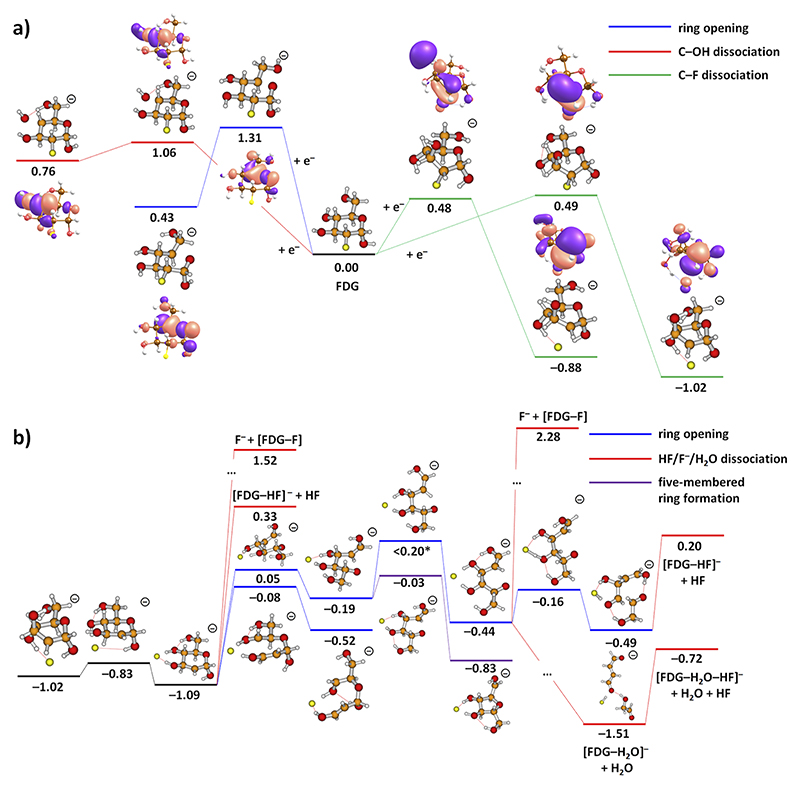
Simplified potential energy surface for (a) processes immediately after electron attachment to FDG and (b) subsequent reactions. In (a), orbitals with the odd electron are shown for each step. Reaction energies are given in eV with respect to isomer **IV** of neutral FDG (Fig. 3) as calculated at the CCSD/aug-cc-pVDZ//B3LYP/aug-cc-pVDZ level along with the correction for the aug-cc-pVDZ(C, O, F)TZ(H)+ basis, see the Experiment and Theory section. Due to convergence problems, the upper bound for the transition state energy marked with “*” was obtained through interpolation.

In Fig. 4(a), we analyze the initial steps after electron attachment (see Fig. S1 for two further pathways). As the electron is weakly bound, the optimized structure of the FDG anion does not differ considerably from the one in the neutral state. Upon electron attachment, we have not found any low-lying reaction channels concerning dissociation or transfer of H and OH groups or ring opening. As an example, two such pathways are shown on the left-hand side in Fig. 4(a). Pre-dissociation of an OH group requires 1.06 eV with respect to the energy of the neutral FDG molecule. Ring opening along a C–O bond is more demanding with 1.31 eV. In both cases, the electron localizes on the σ* orbital of the breaking bond.

Dissociation of the C–F bond to form F^−^ was calculated to proceed over a barrier of ~0.5 eV with respect to the neutral FDG molecule. However, the barrier is within reach given the average thermal energy of the FDG molecule (0.52 eV at 383 K), see the right-hand side in Fig. 4(a) with two possible C–F dissociation pathways. In the transition state, the electron is localized in the σ* orbital of the C–F bond; in the final structure, F^–^ is formed and the spin density is localized predominantly in a 2*p* orbital of the carbon atom; a similar charge–spin–density configuration was found in bromoadenine.^38^ Although the C–F dissociation reaction lies high in energy, it represents a plausible initial point for further fragmentation as the lowest-energy pathway found (note that the calculated value of the reaction barrier is influenced by the complicated electronic structure of the dipole-bound state). The proposed F^−^ formation also suggests that the fluorine atom could leave the molecule, e.g., in the form of HF. This is indeed confirmed experimentally, see below.

Figure 4(b) proposes further reaction steps in FDG^−^. Due to the high-dimensional potential energy surface of the molecule, the suggested pathways represent only a subset of possible reactions, and we cannot exclude that more favorable pathways exist. However, we present a set of energetically feasible reactions that are closely linked to experimental observations as discussed further.

After F^−^ is formed with a reaction energy of −1.02 eV, it might move to the side of the ring over a small barrier of −0.83 eV compared to the energy of the neutral FDG molecule. Already from this structure, the fluorine atom might dissociate as F^−^or HF with the overall reaction energy of 1.52 and 0.33 eV, respectively. Alternatively, ring opening may take place, e.g., along the C_5_–O or C_3_–C_4_ bonds (see Fig. 1 for atom numbering) with respective barriers of 0.05 and −0.08 eV.

We use the C_5_–O dissociation pathway to illustrate further possible steps. After the bond is broken, the ring might re-form to a five-membered ring of particular stability (−0.83 eV). Alternatively, rotation along the C_4_–C_5_ bond might take place, forming a molecule with a relative energy of −0.44 eV. This molecule is metastable and might further break along a C–C bond over a barrier of −0.16 eV or undergo further rearrangement and dissociation reactions. Both HF and H_2_O might dissociate within available energy; dissociation of F^−^, on the other hand, is more demanding and requires energy of more than 2 eV. Also, spontaneous C–C bond breaking is observed in the calculations.

Note that F^−^ formation as the initial step suggested in Fig. 4 provides enough energy for the following reactions to take place. Even after the dissociation of HF and/or H_2_O, the energy content might be high enough to initiate further molecular decomposition. We note that for FDG, the DEA process is considerably more severe than for other per-fluorinated aromatic compounds studied previously.^39,40^ DEA to molecules like pentafluorophenol and pentafluoroaniline^39^ predominantly leads to the abstraction of a single HF molecule from the intact aromatic ring, while for FDG, the less stable aliphatic ring tends to break up, leading to a variety of fragment anions with similar intensity.

**Fig. 5:**
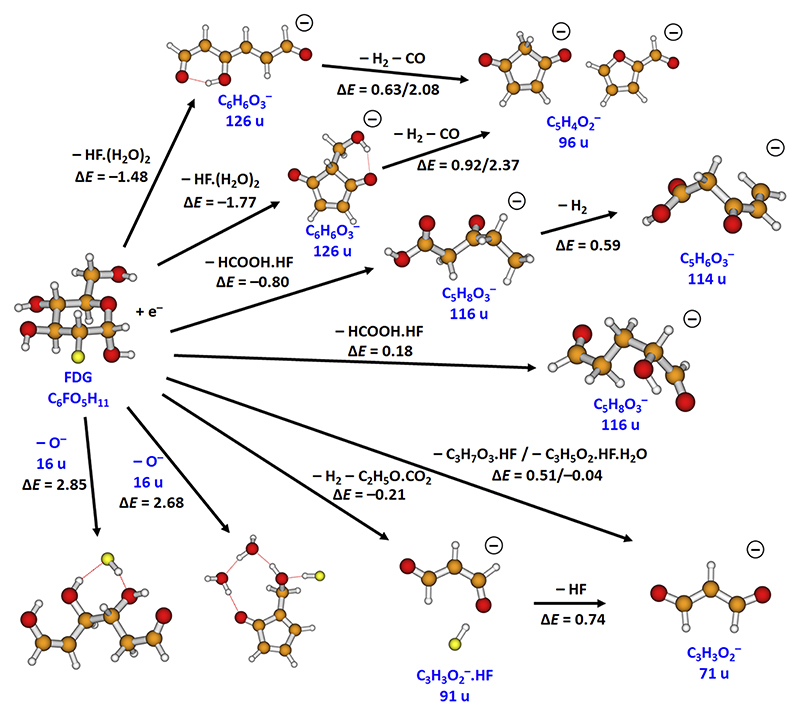
Suggested pathways producing the anions in Table I as retrieved from high-temperature MD. For the formation of the ion with 96 u from 126 u and the ion with 116 u from FDG, only the first dissociation step was observed in the MD run; O^−^ formation was not observed in MD. Reaction energies are given in eV with respect to isomer **I** of neutral FDG (Fig. 3) as calculated at the CCSD/aug-cc-pVDZ//B3LYP/aug-cc-pVDZ level.

To assign possible resulting anions from the experiment (Fig. 2), we used molecular dynamics at a high temperature, speeding up the dissociation reactions by effectively surpassing possible barriers. The results are shown in Fig. 5. Experimentally, the fragment with a mass of 126 u is observed as the second most abundant fragment anion at an electron energy of ~0 eV. In addition, two underlying resonances can be deduced from the tail as well as a very weak feature at 0.8 eV. Based on the MD results, we assign the fragment with a mass of 126 u as C_6_H_6_O_3_^−^, which might be formed through evaporation of neutral HF(H_2_O)_2_. Two different structures were observed in MD runs (Fig. 5), both formed with a reaction energy below −1.4 eV. In the case where the evaporating molecules do not remove enough energy, the anion might dissociate further (see below).

The experimental anion efficiency curves for ions with 116, 114, and 96 u are similar to that of the ion with 126 u, i.e., they are characterized by a peak near 0 eV with a tail that extends to around 1 eV. The fragment anion with a mass of 116 u is assigned as C_5_H_8_O_3_^−^. In molecular dynamics, this anion is not formed most probably because it requires a considerable reorganization that might not be reached during a high-temperature MD (as dissociation is preferred instead) or due to the semi-empirical PM6 method. Two possible low-energy pathways suggested by density functional theory (DFT) calculations follow dissociation of HCOOH⋅HF from FDG^−^ (Fig. 5). The anion with a mass of 114 u might be then formed by subsequent evaporation of H_2_ from the anion with a mass of 116 u. This last dissociation is endothermic (the overall reaction energy being exothermic), and the rate of this reaction will, thus, depend on the energy that is carried away by HF and HCOOH in the first two steps and the thermal energy of the anion. The fragment anion with a mass of 96 u is assigned as C_5_H_4_O_2_^−^ and might be produced, e.g., through H_2_ and CO evaporation from the anion with a mass of 126 u. If this pathway is followed, a five-membered ring has to be formed, which explains why this reaction channel was not observed in the high-temperature MD due to entropic demands.

The anion efficiency curves of ions with masses of 91 and 71 u show a rather similar shape. Compared to the heavier anions discussed before, the tail of the peak at 0 eV is more pronounced, indicating a higher relative intensity of the underlying resonances above zero eV. In addition, for the mass of 71 u, the tail extends to almost 2 eV, and an underlying resonance at 1.2 eV appears. Comparing all yield curves, the ion with a mass of 71 u exhibits the highest yield. The intensity of the second peak at 0.4 eV is higher with respect to the intensity of the other efficiency curves, even at 0 eV. The anion with a mass of 91 u is assigned as C_3_H_3_O_2_^−^⋅HF, which might be formed, e.g., after dissociation of H_2_ and C_2_H_5_O_3_⋅CO_2_, as observed in MD. This anion is also the only fragment for which we predict the presence of the HF molecule that otherwise leaves the anion readily. The hydrogen bond of HF with the anion is quite strong with an interaction energy of about 0.7 eV. The anion at 71 u with the highest intensity could be then produced through evaporation of HF, forming C_3_H_3_O_2_^−^. This explains why the ion dominates at higher electron energies than at lower energies. Alternatively, the ion with 71 u might be produced directly without forming C_3_H_3_O_2_^−^⋅HF along two other reaction pathways (Fig. 5).

The anion efficiency curve for O^−^ shown in Fig. 2 exhibits strong resonances at higher energies between 4 and 12 eV. At energies above 6 eV, the resonance positions observed in our current study are near that of O^−^ reported by Ptasińska *et al*.^26^ for DEA to deoxyribose in the gas phase (7.32, 9.63, and 12.24 eV). The peak at 4.5 eV is close to O^−^ resonances from other DEA studies that reported O^−^ formation.^19,41–43^ In most of these studies, a peak was reported within the energy range of 4.4–5.0 eV. The theoretical threshold for O^−^ formation was found to lie below 3 eV (Fig. 5).

Furthermore, in previous electron attachment studies of several fluorinated compounds, the formation of the F^−^ anion was not detected. For instance, Kopyra *et al*.^43^ and Rackwitz *et al*.^44^ did not report the formation of F^−^ within their studies of gemcitabine and 2-fluoroadenine, respectively. In FDG, we also have not observed any F^−^ anion yield within the detection limit of the apparatus. Our calculations (Fig. 4) show that F^−^ dissociation lies high in energy. Dissociation of an HF molecule, on the other hand, is energetically more feasible. We suggest that before the dissociating F^−^ anion leaves the molecule, it captures a neighboring proton, e.g., from an OH group, and leaves as HF.

Finally, we compare our results to those for native glucose.^27^ In contrast to the present FDG measurements, a considerably higher number of fragments were observed in Ref. 27, which can be ascribed to different experimental setups used (electron source without monochromatizing element; thus providing an electron current, which was more than a factor 10 higher than in the present experiment). However, the attachment characteristics of both molecules seem similar, i.e., for the majority of fragments electron attachment at ~0 eV is observed with the most prominent fragment at 71 u. The authors in Ref. 27 concluded that many fragment anions were produced through evaporation of water molecules, which is an important reaction also for FDG. We suggest that the initial electron attachment process occurs analogously as well. As shown in Figs. 3(c) and 3(d), the electron attachment at 0 eV can be again assigned to a dipole-bound state with the electron positioned next to the CH_2_OH group for the selected isomer with a high dipole moment.

## IV Conclusions

We have investigated dissociative electron attachment to FDG, a potential radiosensitizer known for the inhibition of glycolytic pathways. Our study demonstrates that LEEs with energies as low as 2 eV may decompose the molecule. Seven anions were observed upon electron attachment with the most abundant fragment anion observed at mass 71 u and assigned as C_3_H_3_O_2_^−^. Our calculations suggest that the first pivotal step in electron attachment to FDG is the formation of a weakly bound dipole-state. This represents the only pathway to bind an electron to FDG since no low-lying valence bound states exist. Another pivotal step is found in the dissociation dynamics, corresponding to C–F bond dissociation, forming F^−^ and a radical with an electron on the CH moiety. The dissociation proceeds over a barrier below the thermal energy of the ion. After F^−^ is formed, the gained energy supports further reactions, including ring opening, formation of a five-membered ring, and dissociation of, e.g., HF and H_2_O. We rationalize the lack of observed F^−^ channel by the higher dissociation energy compared to that of HF dissociation.

Thus, it seems that the modification of the native monosaccharide by a fluorine atom does not considerably change the anion characteristics. Previously, it was pointed out that the major advantage of FDG for possible application in the treatment of tumors relies on the similarity between the chemical properties of the fluorine at the C_2_ position in FDG and that of the hydroxyl group in glucose.^10,15^ However, the corresponding electron affinities point to a substantial difference since the fluorine atom has a much higher electron affinity (3.40 eV^45^) compared to OH (1.83 eV^46^). Nevertheless, the comparison of the present results with that of glucose indicates that this imbalance of electron affinities does not have a significant effect on the characteristics of anion formation. In this context, neutral HF formation from the FDG anion seems to act as a counterbalance to the much higher electron affinity.

Our experiments were performed in the gas phase. In the liquid phase, one can expect that formation of small ions, such as F^−47,48^ and OH^−49^, will be more favored. On the other hand, the yield of small neutral species, such as HF, will be suppressed. The next step in understanding dissociation dynamics in FDG^−^ under more complex conditions would be, thus, the study of DEA to microsolvated FDG.

## Supplementary Material

SI

Source data

## Figures and Tables

**Table 1:** Summary of the resonance positions, experimental thresholds, and calculated thermodynamic thresholds for the fragment anions formed upon electron attachment to FDG. Theoretical thresholds are calculated at the CCSD/aug-cc-pVDZ//B3LYP/aug-cc-pVDZ level.

		Resonance positions (eV)						Threshold (eV)	
Mass (u)	Anion	1	2	3	4	5	6	Expt.	Theory
126	C_6_H_6_O_3_^−^	0	0.2	0.4	0.8	…	…	~0	−1.77
116	C_5_H_8_O_3_^−^	0	0.2	0.4	…	…	…	~0	−0.80
114	C_5_H_6_O_3_^−^	0	0.2	0.4	…	…	…	~0	−0.21
96	C_5_H_4_O_2_^−^	0	0.2	0.3	0.5	…	…	~0	−0.85
91	C_3_H_3_O_2_^−^.HF	0	0.2	0.4	0.6	…	…	~0	−0.21
71	C_3_H_3_O_2_^−^	0	0.2	0.4	0.7	1.0	1.2	~0	−0.04
16	O^−^	4.5	7.1	9.2	12.1	…	…	~3.0	2.68

## Data Availability

The data that support the findings of this study are available within the article and its supplementary material.
