# Supplementary material for: Electron attachment to fluorodeoxyglucose: Dissociation dynamics in a molecule of near-zero electron affinity
Source: J Chem Phys. Author manuscript; Available in PMC 2024 Dec 12. (PMC7617131; doi:10.1063/5.0101726)
Supplement: SI [file EMS193762-supplement-SI.docx]

**Supporting Information**

**for**

**Electron Attachment to Fluorodeoxyglucose: Dissociation Dynamics in a Molecule of Near-Zero Electron Affinity**

Eugene Arthur-Baidoo,^a,b^ Milan Ončák,^a*^ Stephan Denifl^a,b *^

*^a^ Institut für Ionenphysik und Angewandte Physik, Leopold-Franzens Universitat Innsbruck, Technikerstrasse 25, A-6020 Innsbruck, Austria.*

*^b^ Center for Molecular Biosciences Innsbruck, Universität Innsbruck, Technikerstrasse 25, A-6020 Innsbruck, Austria*

*Correspondence: Milan.Oncak@uibk.ac.at; Stephan.Denifl@uibk.ac.at

**TABLE SI** – Channels observed in PM6 molecular dynamics of FDG^–^ recalculated as exothermic at the B3LYP/aug-cc-pVDZ level. The stoichiometry and mass of the ion are given as well as frequency of observation (i.e., in how many MD runs was the dissociation observed).

| ion | mass | freq. | 1. step | 2.step | 3. step | 4. step |
| --- | --- | --- | --- | --- | --- | --- |
| C_6_FH_9_O_4_^–^ | 164 | 20 | -H_2_O |  |  |  |
| C_6_FH_7_O_3_^–^ | 146 | 8 | -H_2_O | -H_2_O |  |  |
| C_3_H_3_O_2_^–^ | 71 | 4 | -HF | -C_3_H_7_O_3_ |  |  |
| C_3_H_3_O_2_^–^ | 71 | 3 | -H_2_O | -HF | -C_3_H_5_O_2_ |  |
| C_6_FH_9_O_5_^–^ | 180 | 2 | -H_2_ |  |  |  |
| C_6_H_8_O_4_^–^ | 144 | 2 | -HF | -H_2_O |  |  |
| C_6_H_8_O_4_^–^ | 144 | 2 | -H_2_O | -HF |  |  |
| C_5_FH_9_O_3_^–^ | 136 | 2 | -CO | -H_2_O |  |  |
| C_5_FH_7_O_2_^–^ | 118 | 2 | -H_2_O | -CH_2_O_2_ |  |  |
| C_3_FH_3_O_2_^–^ | 90 | 2 | -H_2_O | -C_3_H_6_O_2_ |  |  |
| C_3_H_2_O_2_^–^ | 70 | 2 | -H_2_O | -C_3_H_6_O_2_ | -HF |  |
| C_5_FH_9_O_3_^–^ | 136 | 1 | -H_2_O | -CO |  |  |
| C_6_FH_5_O_2_^–^ | 128 | 1 | -H_2_O | -H_2_O | -H_2_O |  |
| C_6_H_6_O_3_^–^ | 126 | 1 | -H_2_O | -H_2_O | -HF |  |
| C_6_H_4_O_3_^–^ | 124 | 1 | -H_2_O | -HF | -H_2_O | -H_2_ |
| C_5_FH_7_O_2_^–^ | 118 | 1 | -H_2_CO_2_ | -H_2_O |  |  |
| C_5_H_5_O_3_^–^ | 113 | 1 | -HF | -H_2_O | -CH_2_OH |  |
| C_3_FH_5_O_3_^–^ | 108 | 1 | -H_2_O | -C_3_OH_4_ |  |  |
| C_5_FH_5_O^–^ | 100 | 1 | -H_2_O | -H_2_O | -CH_2_O_2_ |  |
| C_3_FH_4_O_2_^–^ | 91 | 1 | -H_2_O | -H_2_O | -C_3_H_3_O |  |
| C_4_FH_5_O^–^ | 88 | 1 | -H_2_CO | -H_2_O | -H_2_ | -CO_2_ |
| C_4_H_4_O_2_^–^ | 84 | 1 | -H_2_O | -C_2_FH_3_O | -H_2_O |  |
| C_3_H_3_O_2_^–^ | 71 | 1 | -H_2_ | -C_3_O_3_H_5_ | -HF |  |
| C_3_H_3_O_2_^–^ | 71 | 1 | -HF | -H_2_O | -C_3_O_2_H_5_ |  |
| C_3_FH_4_O_2_^–^ | 71 | 1 | -H_2_O | -C_3_H_5_O_2_ | -HF |  |
| C_3_H_2_O_2_^–^ | 70 | 1 | -HF | -C_3_H_6_O_2_ | -H_2_O |  |
| C_3_H_2_O_2_^–^ | 70 | 1 | -H_2_O | -HF | -C_3_H_6_O_2_ |  |
| C_3_H_2_O_2_^–^ | 70 | 1 | -C_3_H_6_O_2_ | -H_2_O | -HF |  |
| C_3_H_4_O^–^ | 56 | 1 | -HF | -C_3_H_4_O_3_ | -H_2_O |  |
| CHO_2_^–^ | 45 | 1 | -H_2_O | -C_5_FH_8_O_2_ |  |  |

**
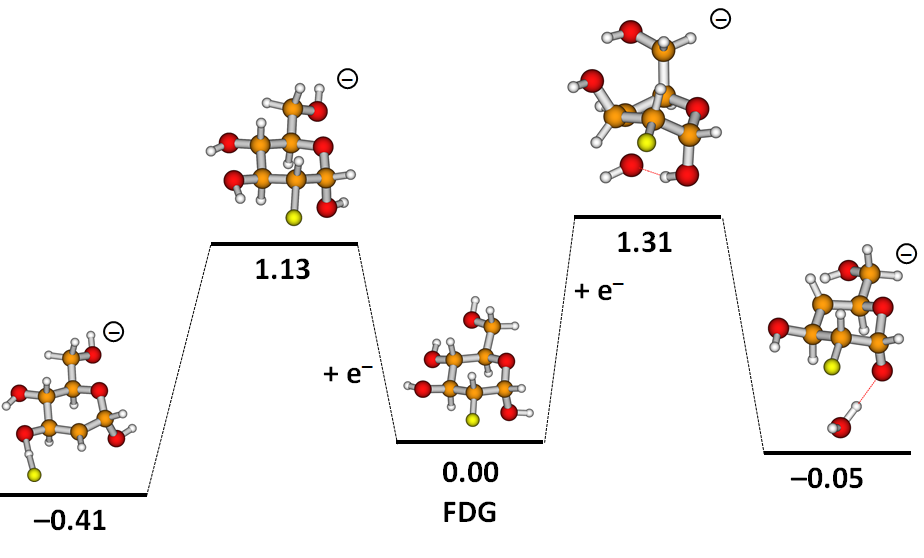
**

**FIG. S1** – Simplified potential energy surface for two reactions after electron attachment to FDG: F dissociation (left) and water formation (right). Reaction energies are given in eV with respect to isomer **IV** of neutral FDG (Figure 3) as calculated at the CCSD/aug-cc-pVDZ//B3LYP/aug-cc-pVDZ using correction for the aug-cc-pVDZ(C,O,F)TZ(H)+ level, see the Methods section.

**Cartesian coordinates of optimized structures (in Å) along with their energies including the zero-point correction (in a.u.) at the B3LYP/aug-cc-pVDZ level**

FDG, I

E = -711.126865

O -0.797765 -1.091410 -0.557851

C -1.191341 0.110300 0.148179

C -0.191221 1.232274 -0.145929

C 1.229650 0.774876 0.188462

C 1.529896 -0.525796 -0.540993

C 0.483981 -1.597224 -0.217150

O -0.562698 2.367049 0.626860

C -2.601787 0.431965 -0.325929

O -3.523553 -0.601554 0.000478

O 2.106522 1.828083 -0.216821

F 2.816089 -0.971981 -0.186912

O 0.603082 -1.931995 1.143437

H -0.227573 1.477276 -1.222752

H 1.307940 0.609486 1.273555

H 1.544409 -0.358133 -1.626247

H 0.619122 -2.479018 -0.859456

H -1.207025 -0.090841 1.228514

H -2.951926 1.339236 0.178055

H -2.585578 0.617810 -1.413989

H -3.198532 -1.417307 -0.401642

H 0.138783 3.025227 0.531539

H 3.004066 1.607733 0.061455

H 0.051734 -2.703405 1.324519

FDG-, I

E = -711.120330

O -0.806466 -1.067564 -0.576409

C -1.187676 0.119606 0.146601

C -0.179647 1.231079 -0.148115

C 1.240824 0.765847 0.185387

C 1.526878 -0.542650 -0.538033

C 0.467515 -1.601580 -0.214216

O -0.528858 2.387952 0.608684

C -2.599907 0.449796 -0.314809

O -3.525616 -0.586294 0.000671

O 2.112340 1.827015 -0.225390

F 2.807151 -1.011873 -0.186265

O 0.552980 -1.942292 1.141146

H -0.212644 1.464229 -1.228459

H 1.320971 0.604052 1.270532

H 1.541583 -0.378796 -1.625599

H 0.584549 -2.490204 -0.854238

H -1.196100 -0.093147 1.225411

H -2.946896 1.354639 0.197501

H -2.584630 0.643692 -1.402486

H -3.130621 -1.423677 -0.287794

H 0.234327 2.986140 0.567666

H 2.953239 1.768448 0.256330

H 0.128893 -2.811657 1.267021

FDG, II

E = -711.126109

O -0.247512 -1.485258 -0.644662

C -1.098125 -0.538376 0.034910

C -0.575147 0.878324 -0.230718

C 0.883885 1.001702 0.202342

C 1.695956 -0.079911 -0.494677

C 1.110110 -1.471513 -0.231140

O -1.404148 1.805197 0.472210

C -2.516662 -0.764543 -0.493770

O -3.499029 -0.130221 0.312256

O 1.312379 2.314771 -0.164932

F 3.030577 -0.015916 -0.056354

O 1.272060 -1.762958 1.135625

H -0.631728 1.078107 -1.315235

H 0.954254 0.867426 1.292211

H 1.713200 0.100208 -1.577900

H 1.602434 -2.224954 -0.861917

H -1.088725 -0.733093 1.116947

H -2.732104 -1.839872 -0.468291

H -2.577171 -0.429454 -1.544513

H -3.231638 0.792651 0.433667

H -1.010711 2.683039 0.375554

H 2.207795 2.453360 0.167587

H 1.049102 -2.690518 1.283406

FDG-, II

E = -711.120113

O -0.230371 -1.483265 -0.654554

C -1.084225 -0.560553 0.043020

C -0.589295 0.864475 -0.229963

C 0.868862 1.019794 0.196842

C 1.696968 -0.061309 -0.490719

C 1.132577 -1.462710 -0.225886

O -1.433178 1.789626 0.463951

C -2.503371 -0.813459 -0.467051

O -3.492478 -0.126960 0.295000

O 1.262723 2.338238 -0.183302

F 3.032728 0.013404 -0.058751

O 1.283560 -1.768101 1.130666

H -0.651311 1.052829 -1.316147

H 0.943763 0.893138 1.286270

H 1.711224 0.117870 -1.575062

H 1.632695 -2.209695 -0.860414

H -1.050730 -0.760480 1.123552

H -2.719995 -1.886392 -0.381423

H -2.560745 -0.536900 -1.535526

H -3.169424 0.777287 0.434722

H -1.001217 2.658460 0.425818

H 2.063077 2.595897 0.305271

H 1.256968 -2.736371 1.250158

FDG, III

E = -711.124190

C -0.218982 1.207112 -0.172426

C -1.184997 0.042601 0.081181

C 1.204169 0.793206 0.205282

H -0.230380 1.461453 -1.247640

O -0.644677 2.325483 0.598154

C 1.578114 -0.486777 -0.525096

O 2.057604 1.883119 -0.153383

H 1.248484 0.617011 1.290680

C 0.553386 -1.593659 -0.258228

H 1.631935 -0.305500 -1.606909

F 2.865125 -0.890416 -0.122316

O -0.727518 -1.123068 -0.636009

H 0.737866 -2.459466 -0.910295

O 0.633902 -1.952108 1.100788

C -2.594532 0.341767 -0.391879

H -1.213766 -0.172024 1.158864

O -3.467191 -0.649842 0.154227

H -2.870231 1.347603 -0.042034

H -2.614772 0.322645 -1.494363

H -4.366464 -0.466936 -0.139034

H 0.053970 2.991854 0.548038

H 2.952983 1.684791 0.147514

H 0.044342 -2.701867 1.252806

FDG-, III

E = -711.114231

C -0.197751 1.207675 -0.182528

C -1.184328 0.066888 0.092714

C 1.226044 0.788745 0.201395

H -0.209080 1.434342 -1.264869

O -0.593239 2.356907 0.559544

C 1.580108 -0.504025 -0.520201

O 2.081437 1.871530 -0.166915

H 1.267547 0.620813 1.287762

C 0.528173 -1.590832 -0.271609

H 1.644172 -0.320631 -1.601999

F 2.846905 -0.964263 -0.114356

O -0.747471 -1.107909 -0.631061

H 0.699113 -2.451065 -0.937879

O 0.598932 -1.988314 1.084039

C -2.598622 0.373843 -0.359010

H -1.196375 -0.145658 1.171430

O -3.468506 -0.627222 0.166813

H -2.873227 1.373116 0.014374

H -2.629348 0.387036 -1.461502

H -4.345564 -0.529341 -0.241767

H 0.171779 2.953943 0.564505

H 2.870509 1.888070 0.406003

H -0.112644 -2.625959 1.229220

FDG, IV

E = -711.123341

C 1.158447 -1.433212 -0.210919

O -0.209471 -1.512203 -0.588150

C -1.070753 -0.582628 0.094517

C -0.608068 0.859818 -0.165892

C 0.875343 1.041667 0.177037

C 1.697452 -0.030404 -0.516967

C -2.460701 -0.882100 -0.460169

O -3.392240 0.014917 0.165748

O -1.311586 1.811787 0.628026

O 1.341134 2.312368 -0.259987

F 3.036609 0.040524 -0.121540

O 1.356861 -1.697877 1.156261

H -0.740737 1.085194 -1.240004

H 0.999165 0.940182 1.266716

H 1.678354 0.129095 -1.604129

H 1.663214 -2.180070 -0.839046

H -1.053817 -0.772617 1.177889

H -2.719835 -1.928999 -0.241988

H -2.452433 -0.737837 -1.552121

H -4.270143 -0.130350 -0.203500

H -2.258417 1.620900 0.548395

H 0.773448 2.979663 0.147764

H 1.223832 -2.640657 1.313050

FDG-, IV

E = -711.122000

C 1.173019 -1.428870 -0.222859

O -0.188398 -1.501145 -0.612672

C -1.062078 -0.594424 0.091238

C -0.612103 0.847900 -0.182711

C 0.860980 1.039457 0.188584

C 1.701918 -0.016153 -0.507165

C -2.460828 -0.907408 -0.425739

O -3.372107 0.016355 0.181711

O -1.341580 1.816028 0.568170

O 1.315510 2.333864 -0.211021

F 3.044895 0.076792 -0.104669

O 1.372588 -1.719504 1.136894

H -0.724831 1.044929 -1.264837

H 0.969303 0.922794 1.278083

H 1.690611 0.153585 -1.593189

H 1.684527 -2.165465 -0.862122

H -1.012525 -0.788706 1.171667

H -2.723999 -1.945575 -0.166858

H -2.476537 -0.808964 -1.523956

H -4.278971 -0.167886 -0.132671

H -2.282042 1.567582 0.505408

H 0.644859 2.957213 0.100458

H 1.211997 -2.668417 1.277286

FDG, V

E = -711.145543

C -1.731479 0.121437 -0.314485

C -3.165421 -0.217824 0.070117

O -3.911015 -0.367916 -1.144523

C -0.826326 0.292063 0.909533

O -0.630705 -0.922834 1.629270

C 0.519741 0.991183 0.604523

O 0.205753 2.341843 0.289023

C 1.352580 0.407457 -0.559670

F 2.431970 1.283931 -0.768172

C 1.870985 -1.000590 -0.313173

O 3.177076 -1.052604 -0.020878

O 1.154183 -1.980774 -0.368440

H -1.317736 0.968115 1.623610

H 1.132918 0.939010 1.519527

H 0.764691 0.400551 -1.485767

H -1.343284 -0.676750 -0.964024

H -1.732876 1.054508 -0.894876

H -3.193449 -1.145870 0.661822

H -3.589215 0.595777 0.684506

H -4.813816 -0.625329 -0.927070

H -0.303789 -1.593949 1.011563

H 1.027021 2.811221 0.093819

H 3.398994 -1.986744 0.133754

FDG-, V

E = -711.196536

C -1.592703 0.213190 -0.367505

C -3.097670 0.115186 -0.186587

O -3.721200 0.024086 -1.486079

C -0.857887 0.225247 0.979647

O -0.995295 -1.012824 1.677497

C 0.627068 0.708909 0.843271

O 0.608356 2.084466 0.661851

C 1.462167 0.006222 -0.186521

F 2.519220 2.666798 -0.659831

C 1.662840 -1.411344 -0.251827

O 2.570621 -1.791586 -1.211855

O 1.116548 -2.291883 0.433335

H -1.312009 0.987260 1.629042

H 1.081388 0.431894 1.830594

H 2.033017 0.653509 -0.859837

H -1.248983 -0.643232 -0.966356

H -1.340310 1.134400 -0.910447

H -3.345919 -0.773002 0.415465

H -3.467939 1.010802 0.343017

H -4.671852 -0.062847 -1.357165

H -0.328695 -1.628043 1.314149

H 1.472093 2.404627 0.082451

H 2.601093 -2.759083 -1.163306

FDG, VI

E = -711.107637

C 1.337218 -0.091042 -0.229346

C 0.411773 -0.016784 1.010058

C -1.030225 -0.533656 0.782007

C -1.708699 -0.108379 -0.532980

C -2.081476 1.354811 -0.681038

O -1.369964 2.282162 -0.358089

C 2.797550 -0.017843 0.205059

O 3.589094 0.294035 -0.940857

O 0.430729 1.281111 1.580852

O -0.947706 -1.962317 0.816658

F -2.923275 -0.834953 -0.633944

H -3.077943 1.525102 -1.144278

H 0.809137 -0.682861 1.790501

H -1.638838 -0.172823 1.626421

H -1.086655 -0.413208 -1.388699

H 1.140207 0.781189 -0.870911

O 1.087179 -1.235641 -1.051030

H 2.901040 0.758440 0.979827

H 3.087443 -0.994075 0.632649

H 4.516918 0.319775 -0.682647

H 0.010649 1.890463 0.950681

H -1.816373 -2.326705 0.600496

H 0.792373 -1.968158 -0.491376

FDG-, VI

E = -711.177824

C 1.343054 -0.372928 -0.194951

C 0.429116 0.110315 0.956832

C -1.089204 -0.106027 0.615832

C -1.581813 0.582626 -0.625846

C -1.509133 1.963803 -0.957273

O -0.913298 2.854765 -0.305840

C 2.802387 -0.336363 0.237730

O 3.646153 -0.436398 -0.926563

O 0.739161 1.435743 1.362375

O -1.344644 -1.468811 0.596220

F -3.531968 -1.831739 -0.209396

H -2.028229 2.258360 -1.893969

H 0.596421 -0.536486 1.830759

H -1.625355 0.405275 1.455789

H -2.133362 -0.056251 -1.319282

H 1.232787 0.335180 -1.039731

O 1.001066 -1.685871 -0.616091

H 2.996569 0.608616 0.767148

H 2.982877 -1.188764 0.914129

H 4.559927 -0.486458 -0.627201

H 0.222976 2.062172 0.812138

H -2.495725 -1.695673 0.187568

H 0.084880 -1.864302 -0.277522

glucose

E = -687.095531

C 1.689784 -0.071505 -0.522702

C 1.134281 -1.459723 -0.190241

O -0.240355 -1.523451 -0.562649

C -1.089746 -0.567947 0.095722

C -0.603474 0.868963 -0.169433

C 0.873932 0.999931 0.190643

O 1.324892 -1.717565 1.180035

C -2.479269 -0.849098 -0.469138

O -3.403445 0.067193 0.141483

O -1.298437 1.834687 0.617381

O 1.407197 2.263456 -0.215998

O 3.065593 -0.022147 -0.183912

H -0.723336 1.094585 -1.244741

H 0.985989 0.880391 1.279137

H 1.563428 0.066063 -1.610806

H 1.622454 -2.224545 -0.809626

H -1.089655 -0.742744 1.182275

H -2.756997 -1.890009 -0.245470

H -2.459311 -0.713035 -1.561929

H -4.279124 -0.066153 -0.237253

H -2.247191 1.654694 0.535548

H 0.882827 2.953249 0.210850

H 1.161829 -2.654459 1.342801

H 3.342482 0.900856 -0.260610

glucose-

E = -687.091618

C 1.695853 -0.060050 -0.513858

C 1.146398 -1.458330 -0.205803

O -0.221533 -1.515973 -0.585741

C -1.083353 -0.578320 0.092601

C -0.606757 0.857601 -0.187701

C 0.860652 0.995193 0.199622

O 1.342426 -1.745729 1.157764

C -2.483198 -0.869758 -0.433649

O -3.382240 0.074572 0.160589

O -1.322377 1.840280 0.558436

O 1.388653 2.279484 -0.169354

O 3.074454 0.015787 -0.165372

H -0.708392 1.054814 -1.270442

H 0.955992 0.860754 1.287755

H 1.580291 0.087687 -1.601886

H 1.639371 -2.212035 -0.839161

H -1.050607 -0.756649 1.176827

H -2.767926 -1.901222 -0.170075

H -2.488781 -0.778581 -1.532583

H -4.287778 -0.085116 -0.171898

H -2.267355 1.609893 0.490537

H 0.764852 2.938053 0.165407

H 1.104863 -2.674912 1.310069

H 3.312831 0.951932 -0.202394

TS, F disoc, 1

E = -711.111520

c 1.664665 0.456130 0.115234

f 1.651148 0.535039 -1.484721

c 1.369859 -0.981553 0.431090

c 0.001484 -1.395477 -0.164285

c -0.991943 -0.207194 -0.198807

o -0.663082 0.806537 0.761175

c 0.595120 1.442916 0.570181

o 0.503644 2.561288 -0.294680

o 1.399554 -1.106726 1.866975

o 0.137326 -1.941879 -1.481800

c -2.416245 -0.626524 0.125800

o -3.332513 0.464357 0.023970

h -2.894051 1.215764 0.448264

h -2.744567 -1.392812 -0.588413

h -2.436605 -1.055670 1.143337

h -0.970392 0.228940 -1.210673

h 0.619562 -1.278314 -2.001634

h -0.394581 -2.211361 0.452605

h 0.749398 2.247570 -1.182050

h 0.850308 1.838695 1.560261

h 2.676482 0.782198 0.361681

h 2.140599 -1.637714 -0.008253

h 1.846434 -1.951045 2.106971

LM, F disoc, 1

E = -711.167533

c -1.565119 -0.149074 -1.045327

f -1.655542 1.654039 1.451991

c -1.218692 -1.305489 -0.176411

c -0.095865 -0.980001 0.851437

c 0.933062 0.053157 0.305364

o 0.770297 0.352561 -1.088893

c -0.538823 0.879783 -1.410487

o -0.789318 2.126085 -0.849522

o -0.847507 -2.430271 -1.039456

o -0.631569 -0.558627 2.083578

c 2.368254 -0.432490 0.443227

o 3.307576 0.528940 -0.043409

h 2.882613 0.922176 -0.820240

h 2.597247 -0.607377 1.503555

h 2.481153 -1.385133 -0.106757

h 0.804340 0.978131 0.884176

h -1.074092 0.345029 1.921715

h 0.428722 -1.933568 1.051306

h -1.168360 1.995435 0.095336

h -0.484329 1.014489 -2.504503

h -2.595308 0.013519 -1.361246

h -2.097153 -1.599821 0.418832

h -0.647704 -3.174049 -0.455305

TS, F disoc, 2

E = -711.109251

c 1.728146 0.331423 0.185398

f 1.362069 0.058321 1.719397

c 0.783186 1.443716 -0.196528

c -0.677685 0.988419 -0.013342

c -0.839755 -0.548268 -0.130469

o 0.138711 -1.103055 -1.004441

c 1.475200 -1.000605 -0.522016

o 1.824292 -2.094689 0.306485

o 0.931697 1.819319 -1.579522

o -1.244530 1.423481 1.237352

c -2.216066 -0.934381 -0.661883

o -3.259755 -0.512697 0.222734

h -2.939138 0.257339 0.719254

h -2.282229 -2.027191 -0.752824

h -2.356592 -0.493615 -1.664463

h -0.731229 -0.980010 0.878127

h -0.670511 1.067474 1.936409

h -1.267922 1.493195 -0.787609

h 1.665127 -1.812038 1.223100

h 2.097477 -1.074167 -1.419332

h 2.785165 0.604628 0.229201

h 0.965125 2.320976 0.447086

h 1.834622 2.177827 -1.711352

LM, F disoc, 2

E = -711.170548

c 1.841959 0.592627 0.191251

f 1.054060 -2.188947 1.058024

c 0.749699 1.266921 0.945039

c -0.578884 0.469226 0.877404

c -0.776912 -0.147081 -0.540231

o 0.152465 0.386510 -1.493923

c 1.524985 -0.000614 -1.151341

o 1.742252 -1.367152 -1.206326

o 0.529257 2.619086 0.424368

o -0.688560 -0.536339 1.866557

c -2.192246 0.062917 -1.057178

o -3.148965 -0.540893 -0.174396

h -2.697400 -0.759101 0.656266

h -2.303271 -0.398609 -2.049583

h -2.408922 1.142395 -1.149673

h -0.605858 -1.228126 -0.452050

h -0.006354 -1.254686 1.632906

h -1.374612 1.203356 1.083839

h 1.497650 -1.773302 -0.294966

h 2.109692 0.464154 -1.963886

h 2.833889 0.445680 0.615275

h 1.008696 1.407078 2.000511

h 0.356760 2.498008 -0.520759

TS, F disoc, 3

E = -711.097467

o -0.792866 -1.164386 -0.576166

c -1.220279 0.039432 0.092456

c 0.563981 -1.590290 -0.277878

c 1.501320 -0.464769 -0.572475

c 1.163538 0.773523 0.189894

o 2.074869 1.849401 -0.082034

c -0.247375 1.204844 -0.195552

o -0.679515 2.347888 0.564564

c -2.632511 0.337388 -0.375749

o -3.516319 -0.654153 0.154111

o 0.657650 -1.997038 1.078520

h -0.090209 -2.588220 1.235216

h 0.713292 -2.443274 -0.956099

f 3.142438 -0.771984 -0.102849

h 1.651017 -0.314474 -1.642593

h 2.952758 1.431392 -0.055234

h 1.170558 0.548649 1.268392

h -0.264640 1.446889 -1.272904

h 0.074885 2.951925 0.584762

h -2.916742 1.341087 -0.020266

h -2.650140 0.331184 -1.478279

h -4.407172 -0.487623 -0.194616

h -1.238147 -0.124145 1.181125

LM, F disoc, 3

E = -711.151825

o -1.191658 -1.022284 -0.660751

c -1.303128 0.205054 0.086348

c -0.031114 -1.805094 -0.384093

c 1.230852 -1.012301 -0.352751

c 1.202914 0.366027 0.230919

o 2.275714 1.196771 -0.047909

c -0.084303 1.075390 -0.224977

o -0.177163 2.342450 0.427364

c -2.604808 0.868343 -0.313016

o -3.712036 0.138331 0.249157

o -0.200429 -2.501232 0.868795

h -1.061773 -2.940102 0.821123

h -0.019220 -2.546107 -1.200185

f 4.278936 -0.054423 -0.031215

h 2.187399 -1.482611 -0.578412

h 3.272672 0.608224 -0.030147

h 1.081096 0.235221 1.341426

h -0.021975 1.223876 -1.318301

h 0.756452 2.617051 0.484687

h -2.590890 1.900859 0.065336

h -2.674065 0.883401 -1.413531

h -4.524605 0.588708 -0.004229

h -1.333418 -0.015511 1.165338

TS, OH disoc, 1

E = -711.098725

c -0.600041 0.732673 -0.315258

o -1.811268 1.827046 0.535955

c 0.773521 1.078409 0.193182

c 1.706888 0.097369 -0.492676

c 1.294121 -1.366217 -0.227080

o 1.561753 -1.660089 1.143369

o -0.046622 -1.628026 -0.562578

c -1.013502 -0.650165 0.043067

c -2.398327 -1.040448 -0.464515

o -3.413248 -0.348659 0.245486

o 1.166664 2.415262 -0.129469

f 3.066247 0.235278 -0.081522

h 0.403985 2.968217 0.113942

h 0.819899 0.914764 1.282591

h -2.019021 2.531892 -0.109480

h -0.739318 0.975222 -1.374031

h 1.153552 -2.518429 1.313448

h -0.974012 -0.798131 1.132389

h -2.451601 -0.837357 -1.552003

h -2.557014 -2.119256 -0.316584

h -3.056251 0.563401 0.408772

h 1.891518 -2.031104 -0.871497

h 1.697836 0.279281 -1.576267

LM, OH disoc, 1

E = -711.113003

c -0.567898 0.645438 -0.406768

o -2.452191 1.786086 0.693640

c 0.736617 1.129958 0.133670

c 1.777454 0.198431 -0.471500

c 1.447536 -1.286913 -0.180327

o 1.694651 -1.521384 1.207813

o 0.147525 -1.661144 -0.545513

c -0.911341 -0.729896 -0.006691

c -2.266211 -1.216392 -0.514557

o -3.315847 -0.587321 0.176738

o 1.018031 2.486308 -0.231974

f 3.096437 0.442768 -0.000735

h 0.198985 2.959569 -0.022214

h 0.762503 1.022627 1.231372

h -2.673105 2.340765 -0.067104

h -0.821765 0.959126 -1.421803

h 1.282405 -2.371991 1.404979

h -0.889706 -0.835730 1.087575

h -2.316140 -1.035964 -1.610097

h -2.339040 -2.307252 -0.361892

h -3.036384 0.392080 0.378891

h 2.118500 -1.923065 -0.782454

h 1.811509 0.350819 -1.559236

TS, OH disoc, 2

E = -711.093037

o 0.450923 -1.237460 2.006711

c -0.550105 -0.099479 1.008512

c 0.275092 1.087002 0.628341

c 1.087083 0.792817 -0.641529

f 2.343944 1.440871 -0.579890

c 1.306851 -0.706515 -0.915338

o 2.126227 -1.357217 -0.000980

o 0.019979 -1.314709 -1.075024

c -1.017598 -0.981773 -0.115288

c -2.162522 -0.358372 -0.950194

o -3.252854 0.119862 -0.162844

o -0.627147 2.222080 0.433605

h -0.162359 3.057455 0.675205

h 0.981814 1.365407 1.432123

h 0.678360 -0.722037 2.793425

h 1.643943 -1.373945 0.873721

h 1.787337 -0.815874 -1.897733

h -1.324159 0.116591 1.742193

h -1.370583 -1.930808 0.316691

h -1.749132 0.447793 -1.574538

h -2.576871 -1.133211 -1.611943

h -2.941079 0.928296 0.274930

h 0.567401 1.229966 -1.503824

LM, OH disoc, 2

E = -711.139026

o 2.808887 -1.269542 1.841378

c -0.995761 1.037359 0.233176

c 0.487955 1.154579 0.301295

c 1.072891 0.453552 -0.934424

f 2.503718 0.539920 -0.849464

c 0.615380 -1.031230 -1.008465

o 0.941749 -1.781426 0.003646

o -0.906314 -0.842359 -1.202557

c -1.502994 -0.359692 0.009514

c -3.016600 -0.420971 -0.153668

o -3.705025 0.017313 1.029570

o 0.878573 2.539362 0.351607

h 1.842080 2.547361 0.291448

h 0.880347 0.620033 1.182902

h 3.439231 -0.774360 1.306396

h 2.092334 -1.483204 1.172790

h 0.865612 -1.428950 -2.023348

h -1.539989 1.888234 -0.190154

h -1.192653 -1.019049 0.837351

h -3.320514 0.191633 -1.021068

h -3.327538 -1.457376 -0.334547

h -3.188401 0.746900 1.399108

h 0.807841 1.001137 -1.849410

TS, ring opening from FDG

E = -711.091817

o -0.680460 -1.326011 -0.643584

c -1.281292 0.220209 0.234625

c 0.594055 -1.655808 -0.286768

c 1.549028 -0.480796 -0.525667

c 1.153902 0.779697 0.221587

o 2.049906 1.865576 -0.094199

c -0.254427 1.224708 -0.177188

o -0.557972 2.503321 0.444964

c -2.660453 0.388280 -0.288992

o -3.467082 -0.755537 0.046944

o 0.703459 -2.051648 1.104706

h -0.164959 -2.428664 1.298882

h 0.940418 -2.511009 -0.906499

f 2.884108 -0.821792 -0.148093

h 1.588148 -0.281574 -1.605235

h 2.946317 1.593756 0.153191

h 1.179576 0.583870 1.303482

h -0.282295 1.355524 -1.273405

h 0.237540 3.053983 0.342726

h -3.136584 1.293379 0.153525

h -2.622320 0.536468 -1.382715

h -4.396254 -0.471203 0.027631

h -1.234238 -0.071766 1.285027

LM, ring opening from FDG

E = -711.123763

o 0.287315 -1.938732 -0.683350

c -1.409928 -0.360756 0.381122

c 1.467216 -1.500210 -0.259407

c 1.641072 0.008565 -0.523591

c 0.726651 0.963228 0.236538

o 1.133483 2.324513 -0.056134

c -0.748460 0.846756 -0.166964

o -1.468376 2.040073 0.301387

c -2.556599 -0.986623 -0.311581

o -3.844953 -0.362286 0.030720

o 1.677724 -1.730881 1.191264

h 0.885311 -2.243231 1.401914

h 2.344646 -2.001601 -0.746863

f 2.990365 0.429795 -0.201322

h 1.549914 0.179408 -1.605471

h 2.098315 2.327368 -0.008718

h 0.832320 0.783657 1.316275

h -0.809268 0.835632 -1.268697

h -0.866623 2.786635 0.159187

h -2.417965 -0.932377 -1.403228

h -2.667210 -2.041866 -0.031968

h -3.651212 0.574671 0.171641

h -1.212779 -0.623703 1.420024

F- migration to edge, TS

E = -711.165725

f -0.388590 1.550902 2.167425

o 1.836273 -1.550100 1.341523

c 1.629059 -1.459495 -0.075424

o 0.292262 -1.783305 -0.434351

h -0.939443 1.625264 0.976514

c -0.678996 -0.795172 -0.037138

c -0.372420 0.599462 -0.628143

c 1.102193 1.004268 -0.388219

c 2.019738 -0.130045 -0.633281

c -2.034425 -1.313257 -0.523761

o -3.104313 -0.450159 -0.160955

h -2.716914 0.450868 -0.094859

h -2.226113 -2.303064 -0.081806

h -1.984080 -1.438707 -1.622927

h -0.700824 -0.701522 1.059011

h -0.528142 0.562865 -1.721098

h 1.140346 1.311641 0.677854

o 1.462558 2.132629 -1.202491

h 0.794910 2.804592 -1.006202

h 3.024434 0.033611 -1.021859

h 2.236836 -2.271855 -0.492103

h 1.463597 -0.760809 1.764188

o -1.244053 1.562739 -0.072193

F- migration to edge, LM

E = -711.177733

f -3.552295 0.488754 0.212894

o 2.712660 1.275375 0.998865

c 2.026459 1.142777 -0.269339

o 1.865356 -0.217902 -0.623129

h -2.481751 -0.430266 0.276288

c 0.776206 -0.889909 0.031900

c -0.566792 -0.228339 -0.316880

c -0.545807 1.260541 0.122965

c 0.739989 1.894608 -0.294460

c 0.842069 -2.354593 -0.411505

o -0.053519 -3.177155 0.322248

h -0.874334 -2.647257 0.408131

h 1.860400 -2.734115 -0.244039

h 0.635003 -2.403457 -1.497314

h 0.918259 -0.858940 1.126389

h -0.685364 -0.238157 -1.418107

h -0.601960 1.220306 1.240042

o -1.621449 2.015775 -0.379079

h -2.472560 1.517104 -0.151329

h 0.738468 2.944724 -0.585511

h 2.745619 1.536400 -0.999013

h 2.043565 1.325215 1.693360

o -1.588977 -0.968701 0.313466

[FDG-F] after F- migration

E = -611.212161

o 1.811136 -2.101546 0.843228

c 1.532165 -1.330245 -0.329244

o 0.168480 -1.411334 -0.708371

h -1.035701 2.661061 0.199904

c -0.732082 -0.570415 0.020558

c -0.355863 0.888648 -0.262372

c 1.060257 1.166750 0.245268

c 1.996344 0.087544 -0.195934

c -2.148604 -0.931443 -0.439077

o -3.148471 -0.405273 0.421030

h -2.996751 0.548073 0.495065

h -2.249992 -2.023009 -0.415588

h -2.291525 -0.596638 -1.481529

h -0.667972 -0.773461 1.103660

h -0.367735 1.042006 -1.353941

h 0.995066 1.210390 1.352667

o 1.420107 2.470428 -0.240484

h 2.203896 2.783428 0.226246

h 3.058022 0.304348 -0.317517

h 2.071840 -1.854134 -1.127020

h 1.622633 -1.577103 1.631996

o -1.308138 1.748976 0.365954

[FDG-HF]- after F- migration

E = -610.673986

O 2.050982 -1.697237 0.954969

C 1.647162 -1.189343 -0.330001

O 0.275703 -1.507799 -0.583901

C -0.688619 -0.675696 0.091657

C -0.494578 0.816994 -0.271229

C 0.981594 1.175874 0.195129

C 1.925851 0.275664 -0.488129

C -2.087316 -1.141292 -0.316387

O -3.074497 -0.358872 0.335018

H -2.667394 0.557372 0.388494

H -2.234745 -2.198611 -0.041178

H -2.172057 -1.066540 -1.418988

H -0.586732 -0.796488 1.184262

H -0.467674 0.861277 -1.392237

H 0.959634 1.002023 1.293134

O 1.203644 2.556962 -0.049833

H 0.304776 2.921703 0.084911

H 2.396904 0.611625 -1.412911

H 2.196346 -1.793391 -1.062509

H 2.026361 -0.962433 1.579738

O -1.388329 1.668229 0.282628

ring opening along C-C bond after F- migration, TS

E = -711.150883

c 1.203374 -0.246610 0.000965

c -0.140858 -0.773875 -0.441034

c -1.440741 0.968166 0.115969

c -0.710792 1.984213 -0.475421

c 0.752099 2.134132 -0.199802

o 1.543923 1.003988 -0.603754

o 1.047664 2.411708 1.180409

c 2.345740 -1.218531 -0.350374

o 2.189295 -2.478971 0.297228

o -0.555619 -1.854224 0.267416

o -2.683269 0.670828 -0.313149

f -2.967103 -1.750128 0.256141

h -2.911857 -0.284355 -0.032646

h -1.616257 -1.924517 0.233255

h -0.384277 -0.743096 -1.511959

h 1.232514 -2.652471 0.358326

h 2.385943 -1.340682 -1.448138

h 3.302120 -0.790537 -0.015180

h 1.186831 -0.140928 1.098414

h -1.191441 0.608400 1.121777

h -1.068354 2.375823 -1.429039

h 0.199747 2.590091 1.603716

h 1.180064 2.941825 -0.810811

ring opening along C-C bond after F- migration, LM

E = -711.162358

c 1.595563 0.144110 0.149753

c 0.625766 -0.986230 0.023706

c -2.245097 0.569708 -0.286366

c -1.218064 1.283093 -0.763922

c -0.156614 1.903390 0.113888

o 1.146459 1.460191 -0.323752

o -0.353057 1.688898 1.491865

c 2.859002 -0.124958 -0.670873

o 3.542138 -1.296253 -0.216339

o -0.214013 -1.222124 1.046156

o -3.066651 -0.209612 -1.040449

f -2.254129 -2.251647 0.262044

h -2.964577 -1.114246 -0.604320

h -1.105210 -1.732896 0.710462

h 0.315739 -1.323793 -0.970555

h 2.843482 -1.943799 -0.023500

h 2.583718 -0.220625 -1.735977

h 3.558466 0.714169 -0.569431

h 1.869184 0.247044 1.211559

h -2.428719 0.519716 0.790510

h -1.012333 1.291236 -1.835522

h -0.479978 0.729456 1.621110

h -0.094946 2.995084 -0.009700

ring opening along C-O bond after F- migration, TS

E = -711.147058

o -1.841856 0.542117 -0.779654

c -0.755074 1.020240 -0.051469

c -2.196838 -1.345589 -0.043760

c -0.976563 -1.733556 -0.561339

c 0.315743 -1.322865 0.056500

c 0.537126 0.200178 -0.296181

o 1.584503 0.760609 0.456608

o 1.353398 -2.141602 -0.415172

c -0.531403 2.501290 -0.460419

o 0.392235 3.166179 0.388465

o -2.449126 -1.120917 1.272087

h -1.607680 -1.065506 1.748549

h -3.120004 -1.490896 -0.599176

h -0.947483 -2.058882 -1.599859

h -0.959104 1.030833 1.041315

h -1.489843 3.033725 -0.391094

h -0.199459 2.522049 -1.514571

h 1.117358 2.521387 0.529936

h 2.439956 0.174174 0.349045

h 0.760624 0.223655 -1.380682

h 0.268715 -1.358412 1.170414

h 2.240541 -1.707590 -0.182061

f 3.425021 -0.821536 0.176613

ring opening along C-O bond after F- migration, LM

E = -711.154963

o -0.368343 1.943336 -0.903897

c 0.503757 1.304642 -0.073703

c -2.837137 0.639717 -0.008767

c -1.941101 -0.118725 -0.667225

c -0.814683 -0.893684 -0.048389

c 0.564774 -0.222224 -0.342648

o 1.589801 -0.779549 0.444143

o -0.868551 -2.214409 -0.543891

c 1.933580 1.921227 -0.337186

o 2.857298 1.519647 0.647146

o -2.889015 0.859977 1.339903

h -2.163850 0.371769 1.757744

h -3.635170 1.176308 -0.520224

h -2.058763 -0.210017 -1.745938

h 0.289127 1.481565 1.002639

h 1.854641 3.015709 -0.304300

h 2.242957 1.614354 -1.351447

h 2.720780 0.547663 0.731591

h 1.589281 -1.828443 0.350789

h 0.759303 -0.363663 -1.423358

h -0.901702 -0.900923 1.062287

h -0.029181 -2.701161 -0.262859

f 1.367547 -3.180099 0.189261

5-membered ring formation, TS

E = -711.151709

o 0.332076 -1.815754 -0.738218

c 1.721813 -0.265396 -0.707607

c -0.664213 -1.217968 0.029323

c -0.654055 0.289883 -0.253121

c 0.810819 0.758004 -0.068377

o 1.081760 2.017507 -0.631971

c 2.767994 -0.875409 -0.057647

o 2.973371 -0.874263 1.285095

c -2.035509 -1.837037 -0.327075

o -3.070754 -1.329423 0.506119

o -1.569191 0.963310 0.579106

h -2.872451 -0.378451 0.633862

h -2.240122 -1.634785 -1.394174

h -1.997793 -2.926431 -0.183713

h -0.510593 -1.375129 1.118884

h -0.898641 0.444016 -1.322474

h -1.438355 1.978440 0.426564

h 0.968116 0.789887 1.036047

h 1.775970 -0.239221 -1.793164

h 3.497356 -1.496818 -0.574312

h 2.302737 -0.304281 1.694980

h 0.319449 2.638325 -0.362192

f -0.951650 3.300107 0.113964

5-membered ring formation, LM

E = -711.169640

o 0.471281 -1.638896 -0.456055

c 1.424023 -0.522448 -0.681833

c -0.725166 -1.127008 0.153409

c -0.744335 0.358828 -0.190931

c 0.744892 0.722231 -0.069633

o 1.140154 1.896859 -0.713154

c 2.770337 -0.863622 -0.166063

o 3.035621 -0.888616 1.179903

c -1.957313 -1.874831 -0.339662

o -3.132132 -1.370724 0.298129

o -1.632285 1.068965 0.635359

h -2.986167 -0.418747 0.458495

h -2.025961 -1.770208 -1.437902

h -1.887246 -2.946836 -0.101724

h -0.669369 -1.233822 1.252750

h -1.014730 0.480820 -1.259782

h -1.409826 2.066224 0.482968

h 0.953780 0.784246 1.024905

h 1.503230 -0.369619 -1.768235

h 3.478153 -1.461288 -0.737350

h 2.363564 -0.355024 1.630118

h 0.460693 2.599097 -0.430125

f -0.766873 3.325062 0.122301

F-

E = -99.870373

F 0.000000 0.000000 0.000000

molecule linearization after ring opening along C-O bond, TS through interpolation

c 2.879636 -1.336718 -0.000235

c 1.569624 -1.068289 -0.191270

c 0.892469 0.223392 0.168814

c -0.610789 0.212620 -0.254581

c -1.291851 -1.119393 0.161245

c -2.817539 -1.048563 -0.225496

o 3.791634 -0.506717 0.567037

o 1.590726 1.292122 -0.455141

o -1.319134 1.286461 0.319987

o -0.752947 -2.188745 -0.491847

o -3.522613 -0.146523 0.597821

f 0.031766 3.241583 -0.250547

h 3.297497 0.297544 0.849187

h 3.322249 -2.288418 -0.296913

h 0.960730 -1.844087 -0.649998

h 0.892312 0.368587 1.269170

h 1.035113 2.146646 -0.386938

h -0.628002 0.262880 -1.359743

h -0.840469 2.181431 0.085446

h -1.252280 -1.216112 1.268051

h -3.258976 -2.044589 -0.090172

h -2.877359 -0.774096 -1.293065

h -2.967344 0.664882 0.606168

molecule linearization after ring opening along C-O bond, LM

E = -711.167113

o -0.696299 -2.183371 -0.148290

o 3.213183 -0.609838 -0.868364

c -1.328258 -1.040694 0.249362

c -0.470838 0.215290 -0.070011

c -2.683510 -0.941003 -0.540578

o -3.531839 0.054509 -0.008425

o -1.236797 1.372577 0.182373

c 0.852559 0.209080 0.758576

o 1.766118 1.182939 0.246152

c 1.505287 -1.145276 0.733825

c 2.568484 -1.460190 -0.046030

h 2.794482 0.280643 -0.682102

h 2.990652 -2.467495 -0.053402

h 1.116870 -1.919956 1.389034

h 0.570447 0.477182 1.793503

h 1.265774 2.060819 0.031835

h -0.178731 0.152087 -1.134579

h -0.661404 2.200631 -0.029328

h -1.582688 -1.030911 1.331568

h -3.205896 -1.903193 -0.459411

h -2.440238 -0.758078 -1.601698

h -2.957657 0.845428 0.079840

f 0.390125 3.167230 -0.267078

[FDG-F] after F dissociation from linearized molecule after C-O ring opening

E = -611.193155

C -2.645057 -0.711597 -0.500223

O -3.520421 0.294113 -0.062595

C -1.288963 -0.708553 0.337110

C -0.373089 0.428924 -0.158234

C 0.865737 0.566735 0.744878

C 1.692857 -0.686881 0.808157

C 2.677675 -1.005881 -0.056040

O 3.125248 -0.253039 -1.089523

O -0.713151 -1.919222 0.131799

O -1.117091 1.650093 -0.162949

O 1.599848 1.693079 0.177348

H 2.677536 0.611867 -1.047957

H 3.221006 -1.947195 0.024696

H 1.467709 -1.401307 1.596939

H 0.513744 0.849743 1.748970

H 2.246360 1.994651 0.828181

H -0.041321 0.177333 -1.179065

H -0.490639 2.372531 -0.305940

H -1.587187 -0.529571 1.389489

H -3.140474 -1.673938 -0.336991

H -2.378463 -0.612375 -1.564001

H -3.058688 1.141593 -0.160847

[FDG-H2O]- after H2O dissociation from linearized molecule after C-O ring opening

E = -634.799497

C -2.948422 -0.568097 0.682330

O -1.688870 -1.181454 0.842592

C -3.622887 -1.016517 -0.600589

C 1.175029 1.091907 -0.055532

C 2.182597 0.123491 -0.006334

C 3.530756 0.421890 -0.252968

C 4.603508 -0.523829 -0.217350

O 4.516869 -1.742380 0.032931

O -4.824908 -1.080307 -0.767971

O -0.078631 0.890172 0.157762

F -1.598385 2.803627 0.017278

H 5.609753 -0.089334 -0.442344

H 3.805883 1.453935 -0.493499

H 1.907286 -0.905792 0.234102

H 1.478585 2.126764 -0.300027

H -0.954185 2.042560 0.069069

H -2.911769 -1.302014 -1.414161

H -3.607607 -0.825730 1.527044

H -2.871530 0.533998 0.639125

H -0.990118 -0.528352 0.605331

[FDG-HF-H2O]- after H2O+HF dissociation from linearized molecule after C-O ring opening

E = -534.318545

C 3.006039 -0.824293 -0.506300

C 3.794059 0.142698 0.362847

O 4.953919 0.453345 0.166924

O 1.855359 -1.314873 0.126217

O 0.415121 0.880823 -0.094042

C -0.819340 1.179336 -0.115294

C -1.916175 0.299910 -0.015828

C -3.247364 0.741768 -0.052469

C -4.405317 -0.087835 0.039921

O -4.436360 -1.332287 0.166990

H -5.375054 0.470715 -0.010579

H -3.437450 1.815297 -0.161389

H -1.717949 -0.769053 0.093064

H -1.077562 2.256937 -0.225263

H 3.218925 0.564181 1.221464

H 3.668617 -1.665849 -0.765438

H 2.775220 -0.287113 -1.447467

H 1.169533 -0.590682 0.089638

HF formation in linearized molecule after C-O ring opening, TS

E = -711.156180

o -0.238126 -2.172489 0.161711

o 3.387951 -0.702313 1.066673

c -1.056418 -1.148833 0.358275

c -0.569897 0.137150 -0.582159

c -2.525825 -1.458408 -0.061857

o -3.435368 -0.454597 0.343798

o -1.456704 1.167921 -0.404787

c 0.908631 0.485638 -0.239980

o 1.265969 1.763035 -0.754916

c 1.855830 -0.535302 -0.807858

c 2.945257 -1.013034 -0.193862

h 2.781459 -0.054417 1.452888

h 3.609648 -1.736553 -0.665891

h 1.670921 -0.883735 -1.824030

h 0.975245 0.507939 0.866559

h 0.720413 2.418313 -0.246898

h -0.637972 -0.252904 -1.613133

h -1.050932 2.005396 0.197692

h -1.044208 -0.729390 1.389869

h -2.826358 -2.393715 0.431736

h -2.546697 -1.623996 -1.155121

h -3.002578 0.389532 0.091755

f -0.464909 2.970868 0.771043

HF formation in linearized molecule after C-O ring opening, LM

E = -711.171904

h -2.012891 -1.432810 -0.762935

o -0.561273 -2.037456 -0.449108

c 0.561189 -1.427952 -0.449503

c 0.377278 0.279038 0.686918

o 1.556246 0.840049 0.689159

c 1.733548 -2.127331 0.244343

o 2.966377 -1.432427 0.157150

c -0.816122 1.011199 0.003804

o -0.660567 2.435299 0.081468

c -2.141893 0.716442 0.652042

c -3.063256 -0.189761 0.277438

o -2.966151 -1.124697 -0.695546

h -4.035812 -0.207920 0.780548

h -2.401911 1.378533 1.479676

h 0.177346 2.678343 -0.348833

h -0.847301 0.707679 -1.057658

f 1.960353 2.568482 -0.927098

h 1.831614 1.805481 -0.242484

h 0.856175 -0.823758 -1.333191

h 0.111934 -0.283427 1.597632

h 1.874872 -3.099614 -0.256480

h 1.449180 -2.338117 1.291928

h 2.772085 -0.516667 0.440438

[FDG-HF]- for HF dissociation after HF formation in linearized molecule after C-O ring opening

E = -610.694647

C 1.123863 -0.922425 -0.479327

C 2.477676 -1.047962 0.219255

O 3.327592 0.072676 0.024304

O 0.281627 -1.879067 -0.342746

C 0.333289 0.709362 0.481209

C -0.973359 0.943180 -0.347109

C -2.222255 0.575325 0.402153

C -2.780246 -0.650652 0.445264

O -2.334256 -1.794190 -0.125452

O 1.182241 1.676461 0.488594

O -1.005865 2.320947 -0.730770

H -1.347407 -1.767744 -0.309969

H -3.723401 -0.802335 0.979511

H -2.717167 1.379820 0.948303

H -0.108914 2.632931 -0.490463

H -0.923039 0.330863 -1.264366

H 1.178279 -0.376696 -1.446530

H 0.198066 0.112004 1.402712

H 2.998009 -1.927069 -0.199441

H 2.302543 -1.252417 1.292289

H 2.778517 0.855046 0.247855

C5H8O3-

E = -420.941179

C -1.071329 -0.110435 -0.621270

C -0.400288 -0.642868 0.650643

C 1.130295 -0.485856 0.616816

C 1.594289 0.937649 0.375471

O 2.541035 1.162153 -0.443508

C -2.555024 0.057434 -0.547944

O -3.170694 0.539633 0.420493

H -0.651155 -1.704272 0.813042

H 1.500491 -0.825631 1.613891

H 1.224379 1.722003 1.063687

O 1.718458 -1.315638 -0.383954

H -0.787596 -0.083201 1.515771

H -0.809936 -0.731242 -1.489242

H -3.116508 -0.198401 -1.480539

H -0.592985 0.891445 -0.793673

H 2.335263 -0.695434 -0.829490

C3O2H5.H2O.HF

E = -444.568867

C -2.800819 -0.214226 -0.394842

O -3.897543 0.172485 -0.164900

C -1.510870 0.157593 0.317682

C -0.348789 -0.742560 -0.077087

O 0.807897 -0.300657 0.648605

H -1.691614 0.122331 1.403174

H -0.569613 -1.787808 0.184592

H -0.172136 -0.686652 -1.161725

H 1.580704 -0.857998 0.421630

H -1.299254 1.204465 0.045843

O 3.296271 -0.916667 -0.312122

H 3.368723 0.048820 -0.402130

H 4.125152 -1.210858 0.082582

H 1.530892 1.047352 0.204327

F 2.159668 1.697135 -0.136165

C3O3H7.HF

E = -444.549436

C -0.028097 1.093073 0.453596

C 0.348292 -0.241615 -0.060358

O -0.507617 -1.319649 0.513929

O -1.190147 1.700665 0.121651

C 1.819969 -0.578883 0.192955

O 2.692055 0.162495 -0.651733

H 0.630863 1.703013 1.068694

H 0.142719 -0.335057 -1.137232

H 2.065604 -0.424790 1.259500

H 1.986802 -1.634809 -0.047523

H -1.809007 1.064635 -0.289122

H 2.575887 1.105712 -0.479912

H -0.524122 -1.204987 1.476300

H -1.924456 -1.026063 -0.122154

F -2.659956 -0.581244 -0.568498

CO

E = -113.325300

C -0.000000 -0.000000 -0.648008

O 0.000000 0.000000 0.486006

C3H3O2-

E = -266.601251

C -1.230582 0.302956 0.000002

C -0.000020 -0.392453 -0.000606

O -2.392792 -0.164854 0.000273

H -1.115493 1.421601 0.000640

C 1.230590 0.303034 -0.000403

H 0.000056 -1.486464 -0.000193

H 1.115619 1.421628 -0.000612

O 2.392779 -0.164895 0.000503

C2H5O.CO2

E = -342.935326

C -2.017605 0.139914 -0.091125

C 2.877743 0.006952 -0.551805

O -2.362050 -0.974813 -0.120812

O -1.713499 1.266993 -0.067917

C 1.809750 0.278024 0.445066

H 3.927719 0.161987 -0.306656

O 0.681168 -0.601820 0.309330

H 2.214650 0.231720 1.471214

H 1.381376 1.287861 0.315205

H 1.002774 -1.512260 0.321277

H 2.609203 -0.241528 -1.578670

C3H3O2-.HF

E = -367.080803

C -0.328060 -1.447823 -0.000109

C 0.960878 -0.904513 -0.000149

O -1.474192 -0.891495 0.000600

H -0.349372 -2.562030 -0.000787

C 1.288096 0.486817 0.000527

H 1.794181 -1.612725 -0.002421

F -2.147403 1.459249 -0.000497

H 0.423159 1.186602 0.002041

O 2.442173 0.958140 -0.000122

H -1.810673 0.514869 0.000206

C5H8O3-, iso1

E = -420.967688

C 2.751214 0.993847 0.362210

H 3.423630 0.189847 0.694788

C 1.658185 0.441350 -0.576938

C 0.800447 -0.625511 0.059338

O 1.032544 -1.834539 -0.079264

H 2.136292 -0.002636 -1.463396

H 1.021182 1.276993 -0.915762

C -0.442023 -0.178184 0.819674

H -0.747117 -0.956405 1.528942

C -1.632665 0.138535 -0.089054

O -1.603963 0.252635 -1.295496

O -2.753356 0.362182 0.646251

H -3.465357 0.665273 0.041996

H -0.252622 0.739622 1.396354

H 3.354403 1.757154 -0.161076

H 2.316838 1.467701 1.254836

C5H8O3-, iso2

E = -419.788543

C 1.948149 -1.560100 0.111529

C 1.904706 -0.182921 0.307239

C 0.918950 0.760826 -0.103632

C -0.264775 0.247871 -0.972489

C -1.362302 -0.308582 -0.119779

O -2.142042 0.666979 0.454458

O 0.956346 2.004779 0.164301

O -1.593371 -1.487304 0.111995

H 1.123173 -2.122157 -0.324678

H 2.747883 0.284299 0.829877

H -0.635462 1.116779 -1.532798

H -2.742606 0.188437 1.046730

H 0.061695 -0.549372 -1.655846

H 2.809485 -2.136189 0.453473

C6H6O3-, iso1

E = -457.916515

C 0.708264 0.577354 -0.000456

C 1.851979 -0.226525 -0.000228

C -0.627627 0.131553 -0.000426

H 0.840421 1.663016 -0.000912

C -1.749745 1.004660 -0.000151

O -0.861471 -1.204872 -0.000404

C -3.067692 0.523836 0.000389

H -1.578340 2.080845 -0.000139

H -3.877845 1.286583 0.000709

O -3.405957 -0.707380 0.000433

C 3.168237 0.318858 0.000219

H 1.751993 -1.313647 -0.000038

O 4.244407 -0.316731 0.000407

H 3.206691 1.439976 0.000861

H -1.859244 -1.303327 -0.000051

C6H6O3-, iso2

E = -457.909011

C -1.404597 -0.541644 -0.105732

C 0.098460 -0.372184 -0.418421

C -1.896474 0.755400 0.244716

O -2.019088 -1.631614 -0.174315

C -0.856555 1.719283 0.216767

H -2.939458 0.945317 0.501705

C 0.377204 1.116221 -0.146993

H -0.955803 2.776266 0.467722

O 1.539493 1.616901 -0.206851

C 1.032125 -1.263174 0.404860

H 0.269396 -0.588653 -1.486242

O 2.403997 -1.006775 0.092989

H 0.843831 -1.087193 1.481269

H 0.831713 -2.323585 0.197631

H 2.454134 -0.033659 -0.027854

H2CO2.HF

E = -290.226867

C 1.122945 0.164845 -0.000063

O 0.380259 1.135328 -0.000041

O 0.733386 -1.102990 -0.000001

H -0.250780 -1.133303 -0.000334

H 2.219435 0.252135 0.000606

F -1.828734 -0.105676 0.000059

H -1.156883 0.584479 -0.000088

H2

E = -1.164107

H -0.000000 -0.000000 0.380507

H 0.000000 0.000000 -0.380507

HF(H2O)2

E = -253.318494

H -1.050835 0.346562 -0.031970

F -0.899039 1.297204 0.004483

H 1.073896 0.788842 0.058501

O 1.635969 -0.004394 0.095008

H 2.333326 0.135826 -0.555886

H -1.084654 -1.776114 0.647057

O -0.804801 -1.246473 -0.109176

H 0.170271 -1.163013 -0.044709

HF

E = -100.451508

H 0.000000 -0.000000 -0.833113

F -0.000000 0.000000 0.092568

O-

E = -75.138730

O 0.000000 0.000000 0.000000

[FDG-O], iso1

E = -635.904126

C -1.372072 -1.317052 0.501373

C -2.751489 -1.500413 -0.080107

O -3.305338 -0.700036 -0.811300

C -0.605411 -0.123079 -0.071865

C 0.751173 0.138347 0.619398

C 1.585149 -1.113261 0.724926

C 2.685829 -1.406415 0.006900

O 3.278272 -0.656201 -0.948661

O -1.415046 1.069005 0.072905

O 1.495481 1.121321 -0.108329

F -0.233332 3.280227 0.134372

H 2.821031 0.207354 -0.965452

H 3.217004 -2.345184 0.170459

H 1.290144 -1.841047 1.479496

H 0.530578 0.522863 1.631841

H 1.080453 1.997459 -0.026870

H -0.406030 -0.291344 -1.141162

H -0.799734 2.498048 0.083532

H -1.492075 -1.226770 1.595616

H -3.273988 -2.441498 0.204814

H -2.240982 0.905914 -0.416053

H -0.812444 -2.249326 0.333757

[FDG-O], iso2

E = -635.916802

C -1.153161 1.237212 0.130056

C -1.014289 -0.135212 -0.501376

C -2.250706 -0.664193 -0.530313

C -3.239330 0.295604 0.074841

C -2.606368 1.411973 0.474419

H -2.519360 -1.647811 -0.911808

C 0.281508 -0.740447 -0.933840

O 0.970478 -1.262666 0.214354

O -0.269599 2.061610 0.299525

H -4.303800 0.084854 0.162216

H -3.013857 2.301370 0.945758

H 0.090368 -1.553304 -1.649999

H 0.912815 0.013734 -1.424907

H 1.921214 -0.999591 0.180127

O 3.451746 -0.219925 0.129979

H 4.126862 -0.409133 -0.530732

H 3.251887 0.741996 0.061580

O 2.508904 2.331441 -0.080481

H 1.543339 2.246041 0.050801

H 2.785614 3.051986 0.496642

H 0.521507 -2.762787 0.519185

F 0.142808 -3.642296 0.590155

C5H4O2-, iso1

E = -343.383264

C -1.225046 -0.107100 0.000066

C 0.000316 -1.042987 0.000398

C 1.224839 -0.107591 0.000191

C 0.709611 1.225215 0.000207

C -0.709621 1.225414 -0.000101

O 2.418641 -0.498930 -0.000499

O -2.418576 -0.499168 -0.000457

H 1.350666 2.108826 -0.000502

H -0.000798 -1.696776 -0.885181

H -1.350502 2.109192 0.000756

H -0.000480 -1.694166 0.888006

C5H4O2-, iso2

E = -343.334037

C 0.302573 -0.101782 0.000011

O -0.661090 -1.130988 0.000001

C -1.906078 -0.531942 -0.000002

C -1.770572 0.838424 -0.000007

C -0.381721 1.132691 0.000008

H -2.761989 -1.197450 -0.000026

C 1.671637 -0.461315 0.000016

H -2.593416 1.551426 -0.000015

H 0.097188 2.105830 0.000021

O 2.649012 0.361556 -0.000016

H 1.859809 -1.560807 -0.000015
